# Heterogenicity within the LPS Structure in Relation to the Chosen Genomic and Physiological Features of the Plant Pathogen *Pectobacterium parmentieri*

**DOI:** 10.3390/ijms23042077

**Published:** 2022-02-14

**Authors:** Karolina Ossowska, Agata Motyka-Pomagruk, Natalia Kaczyńska, Agnieszka Kowalczyk, Wojciech Sledz, Ewa Lojkowska, Zbigniew Kaczyński

**Affiliations:** 1 Faculty of Chemistry, University of Gdansk, 63 Wita Stwosza, 80-308 Gdansk, Poland; karolinaoss@wp.pl (K.O.); agnieszka.kowalczyk@ug.edu.pl (A.K.); 2 Intercollegiate Faculty of Biotechnology University of Gdansk and Medical University of Gdansk, University of Gdansk, 58 Abrahama, 80-307 Gdansk, Poland; agata.motyka-pomagruk@ug.edu.pl (A.M.-P.); natalia.kaczynska@ug.edu.pl (N.K.); wojciech.sledz@biotech.ug.edu.pl (W.S.); ewa.lojkowska@biotech.ug.edu.pl (E.L.)

**Keywords:** soft rot *Pectobacteriaceae*, *Pectobacterium parmentieri* SCC3193, lipopolysaccharide, O-antigen chemical structure, pseudaminic acid, biodiversity, virulence

## Abstract

*Pectobacterium parmentieri* is a pectinolytic plant pathogenic bacterium causing high economic losses of cultivated plants. The highly devastating potential of this phytopathogen results from the efficient production of plant cell wall-degrading enzymes, i.e., pectinases, cellulases and proteases, in addition to the impact of accessory virulence factors such as motility, siderophores, biofilm and lipopolysaccharide (LPS). LPS belongs to pathogen-associated molecular patterns (PAMPs) and plays an important role in plant colonization and interaction with the defense systems of the host. Therefore, we decided to investigate the heterogeneity of O-polysaccharides (OPS) of LPS of different strains of *P. parmentieri*, in search of an association between the selected genomic and phenotypic features of the strains that share an identical structure of the OPS molecule. In the current study, OPS were isolated from the LPS of two *P. parmentieri* strains obtained either in Finland in the 1980s (SCC3193) or in Poland in 2013 (IFB5432). The purified polysaccharides were analyzed by utilizing 1D and 2D NMR spectroscopy (^1^H, DQF-COSY, TOCSY, ROESY, HSQC, HSQC-TOCSY and HMBC) in addition to chemical methods. Sugar and methylation analyses of native polysaccharides, absolute configuration assignment of constituent monosaccharides and NMR spectroscopy data revealed that these two *P. parmentieri* strains isolated in different countries possess the same structure of OPS with a very rare residue of 5,7-diamino-3,5,7,9-tetradeoxy-l-glycero-l-manno-non-2-ulosonic acid (pseudaminic acid) substituted in the position C-8: →3)-β-d-Gal*f*-(1→3)-α-d-Gal*p*-(1→8)-β-Pse4Ac5Ac7Ac-(2→6)-α-d-Glc*p*-(1→6)-β-d-Glc*p*-(1→. The previous study indicated that three other *P. parmentieri* strains, namely IFB5427, IFB5408 and IFB5443, exhibit a different OPS molecule than SCC3193 and IFB5432. The conducted biodiversity-oriented assays revealed that the *P. parmentieri* IFB5427 and IFB5408 strains possessing the same OPS structure yielded the highest genome-wide similarity, according to average nucleotide identity analyses, in addition to the greatest ability to macerate chicory tissue among the studied *P. parmentieri* strains. The current research demonstrated a novel OPS structure, characteristic of at least two *P. parmentieri* strains (SCC3193 and IFB5432), and discussed the observed heterogenicity in the OPS of *P. parmentieri* in a broad genomic and phenotype-related context.

## 1. Introduction

*Pectobacterium parmentieri* is a Gram-negative phytopathogenic bacterium from the family *Pectobacteriaceae* [1]. Before vast taxonomic rearrangements, this species used to be known as *Erwinia carotovora* subsp. *carotovora*, *Pectobacterium carotovorum* subsp. *carotovorum* or *Pectobacterium wasabiae* [2,3,4]. The members of this species are responsible for soft rot and blackleg on potato, and cause economically important losses in its production, as well as soft rot on other cultivated crops, vegetables and ornamentals. The disease symptoms on potatoes involve plant wilting, blackening and maceration of the stem in addition to softening and decay of the inner tuber mass during vegetation and storage. Development of the above-listed disorders depends on efficient production and secretion of a wide range of plant cell wall-degrading enzymes (PCWDE), i.e., pectinases, cellulases and proteases [5]. Additional factors that play roles in setting up disease symptoms include motility, siderophores, biofilm and lipopolysaccharides’ (LPSs’) production. The LPS molecules belong to pathogen-associated molecular patterns (PAMPs), being typical markers of microbial infection within the plant tissue. In particular, LPS accounts for bacterial adhesion and successful attachment to plant tissue, crucial processes undertaken during colonization of the host. Furthermore, in spite of the fact that LPS directly triggers plant defense responses, it may additionally sensitize plant tissue, via accelerated synthesis of antimicrobial hydroxycinnamoyl-tyramine conjugates, changes in the gene expression patterns of pathogenesis-related proteins (PRP) and prevention of the hypersensitive reaction (HR) caused by avirulent bacteria [6,7]. Further proof of the role of these molecules in the virulence of *Pectobacteriaceae* was acquired on LPS-deficient strains of *Pectobacterium atrosepticum*, which revealed a lower growth rate, survival and tissue maceration capacity than the corresponding wild-type strains [8].

Previous research has shown high heterogeneity among *P. parmentieri* strains, both on the genomic and phenotypic levels [9,10]. During pangenome-oriented studies performed on 15 *P. parmentieri* strains, Zoledowska et al. [10] observed that the core genome fraction barely exceeded 50%, and suggested that the extended accessory (20.9%) and unique (26.3%) partitions may explain the notable plasticity of this species in adapting to different environmental niches. This fact is confirmed by the ubiquitous presence of *P. parmentieri* under the diverse environmental conditions of, e.g., Finland, Canada, Poland, South Africa, China, New Zealand and Turkey [11,12,13,14,15,16]. It is also worth noting that even though *P. parmentieri* is a recently established species, the strains currently belonging to this taxon have been in the natural environment for decades, they only started to be noticed after significant advancements in the sensitivity of molecular detection methods [3,13].

One of the most broadly studied strains of *Pectobacteriaceae* is *P. parmentieri* SCC3193, which was isolated from potato plants in Finland in the 1980s [17]. For many years, the SCC3193 strain belonged to the *P. carotovorum* species and was used as a model in research focused on revealing the molecular background of virulence in this species. Due to the above-mentioned progress in genome-based classification methods, the SCC3193 strain underwent notable taxonomic rearrangements. At first, it adhered to the *P. wasabiae* species [2], then went on to be reclassified to a newly established *P. parmentieri* species [4].

In the current study, evidence of heterogeneity within the chemical structure of O-polysaccharide (OPS) in the LPS of *P. parmentieri* is provided. The structures of the OPS of two *P. parmentieri* strains, namely a model SCC3193 [2] and IFB5432 [9], were elucidated. These two strains, acquired in different countries and over a period of nearly 30 years, possess the same OPS molecules. It is worth emphasizing that the described OPS structure is significantly different from the one described earlier for another two *P. parmentieri* strains, IFB5408 and IFB5427, isolated in Poland in 2013 from infected potato plants and asymptomatic weeds growing in the potato fields, respectively [18]. This study aimed to elucidate the relationship between the structure of the OPS molecule and the chosen genomic and physiological features of *P. parmentieri.*

## 2. Results and Discussion

### 2.1. The Chemical Structure of OPS of P. parmentieri SCC3193 and IFB5432

The LPS was isolated from *P. parmentieri* SCC3193 cells using phenol-water extraction, purified by ethanol precipitation and hydrolyzed to cleave lipid A. The mixture of carbohydrates was fractionated by size exclusion chromatography (SEC). The obtained high-molecular-weight OPS was analyzed using NMR spectroscopy and chemical methods.

**Figure 1:**
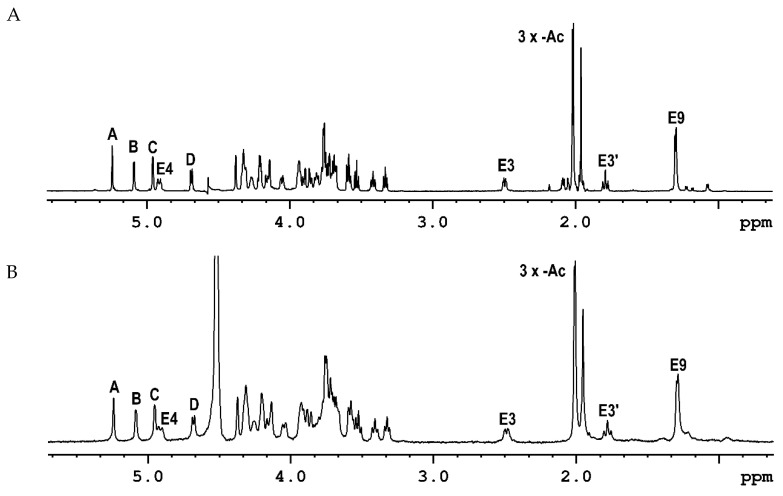
^1^H NMR spectra of the OPS isolated from *P. parmentieri* SCC3193 (**A**) and *P. parmentieri* IFB5432 (**B**).

The results of sugar analysis demonstrated the presence of two different hexoses in the molar ratio ~1:1. The monosaccharides were identified as glucose and galactose by comparing their respective retention times with the standards. The substitution positions of the monosaccharides were determined by methylation analysis. Gas-liquid chromatography-mass spectrometry (GLC-MS) analysis of the partially methylated alditol acetates revealed the presence of three derivatives in the ratio of ~0.7:1.0:2.0, which were identified as: 1,3,4-tri-*O*-acetyl-2,5,6-tri-*O*-methyl-hexitol, 1,3,5-tri-*O*-acetyl-2,4,6-tri-*O*-methyl-hexitol and 1,5,6-tri-*O*-acetyl-2,3,4-tri-*O*-methyl-hexitol. The glycosylation positions were determined based on the arrangement of the acetyl and methyl groups: one residue of →3)-Hex*f*, one residue of →3)-Hex*p* and two residues of →6)-Hex*p*. d configurations for Glc and Gal residues were identified by GLC analysis of the obtained acetylated (*S*)-(+)-butan-2-ol glycosides. Chemical analyses of the OPS of the *P. parmentieri* IFB5432 strain (isolated in the same way as SCC3193) provided practically the same information, indicating that both strains contain the same monosaccharides in the investigated OPS molecules. The ^1^H NMR spectra recorded for OPS isolated from *P. parmentieri* SCC3193 (Figure 1A) and *P. parmentieri* IFB5432 (Figure 1B) are basically identical.

Then, a detailed structural analysis of the OPS of the P. *parmentieri* SCC3193 strain was conducted. The ^1^H NMR of a polysaccharide from *P.* *parmentieri* SCC3193 (Figure 1A) contained several signals in the anomeric region (δ 4.6–5.4), some signals of protons directly linked to carbohydrate-ring carbons in the region of δ 3.3–4.4 and the remaining few signals in the region of δ 1.2–2.6. The HSQC spectrum (Figure S1, Table 1) showed four anomeric cross-peaks (δ 5.241/110.71, 5.088/98.06, 4.959/99.27, 4.689/103.29), which were labeled **A** to **D** according to the decreasing chemical shifts of the anomeric protons.

Moreover, the presence of a “non-anomeric” proton signal (δ 4.911/70.59) in the anomeric region of the ^1^H domain of the HSQC spectrum (Figure S1) suggested *O*-acetylation. The presence of four signals in the anomeric range was consistent with the methylation and sugar analysis results. All constituent residues were identified based on the chemical analyses results and NMR data. The ^3^*J*_H,H_ coupling constant pattern was used to distinguish two galactose and two glucose residues. Residues **D** and **C** possessed a *gluco* configuration, while **B** had a *galacto* configuration. The remaining residue **A** was identified as a β-galactose occurring in the *furano*-ring form. This discovery was also confirmed by the characteristically high chemical shift of the anomeric carbon atom (δ 110.71) and a small value of the coupling constant ^3^*J*_H1,H2_ (1.5 Hz). The lack of carbon atom signals in the region of δ ~83–88 in the ^13^C NMR spectrum (except for residue **A**) also proved that the monosaccharide rings of residues **B**, **C** and **D** possessed the pyranose form. The ^3^*J*_H1,H2_ and ^1^*J*_H1,C1_ coupling constant values were used for anomeric configuration assignment of the remaining three residues. The respective values revealed α-anomeric configuration of **B** (3.7 and 171 Hz), α-anomeric configuration of **C** (3.4 and 172 Hz) and β-anomeric configuration of **D** (7.8 and 161 Hz). Next, 1D and 2D ^1^H and ^13^C NMR spectroscopy (HSQC, TOCSY and COSY spectra; Figures S1–S3) allowed the complete assignment of all ^1^H and ^13^C chemical shifts identified in the OPS (Table 1).

The TOCSY spectrum (Figure S2) showed five spin systems, four of which originated from the anomeric protons described above. In contrast, the fifth spin system, associated with the “non-anomeric” proton signal (δ 4.911), showed couplings that could not belong to the glucose and galactose residues identified earlier. Moreover, the HSQC spectrum showed eight cross-peaks, which were still not used in the analysis, for instance, a CH_2_ group (δ 2.489 and 1.784/34.09), three CH_3_ groups of acetyl groups (δ 1.956/23.48, 2.008/23.21, 2.014/21.76) and a CH_3_ group (δ 1.293/14.19). The above-described findings indicated the presence of an additional component in the structure of the studied OPS, not identified in the earlier stage of structural studies. The detailed structural analysis of the 1D and 2D NMR spectra (DQF-COSY, TOCSY, HSQC and HMBC) allowed for the identification of a sugar derivative containing nine carbon atoms in the molecule -5,7-diamino-3,5,7,9-tetradeoxy-l-glycero-l-manno-non-2-ulosonic acid (pseudaminic acid; Pse) [19,20]. The component is substituted by the *O*-Ac group at C-4 (δ 1.956/23.48) and two *N*-Ac groups at the amino function groups linked to C-5 and C-7 (δ 2.008/23.21 and 2.014/21.76).

The positions of the substitutions of monosaccharides assigned by the methylation analysis results were confirmed by the low-field positions of the linkage carbons: C-3 of unit **A** (δ 85.77), C-3 of unit **B** (δ 78.64), C-6 of units **C** (δ 64.78) and **D** (δ 66.79) and C-8 of unit **E** (δ 74.13) (Table 1) in comparison to the corresponding non-substituted monosaccharides.

**Figure 2:**
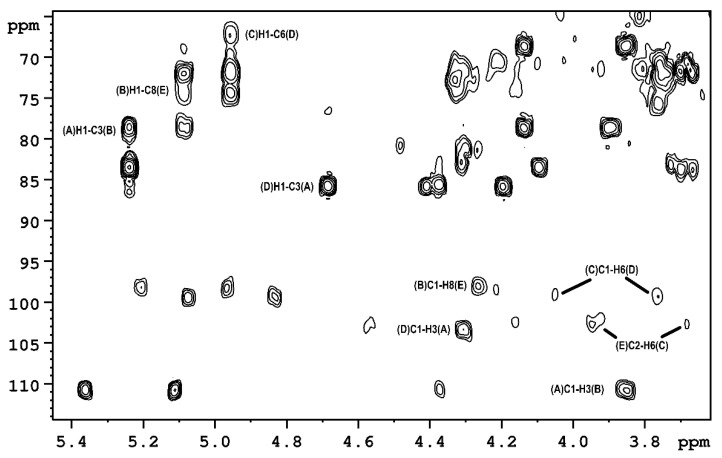
The section of HMBC spectrum of the OPS isolated from *P. parmentieri* SCC3193.

Sequence analyses of the monosaccharides in the O-polysaccharide were performed in a 2D ^1^H,^13^C HMBC experiment (Figure 2), which showed couplings between anomeric carbon atoms and protons attached to glycosylated carbon atoms: (**A**)C1–H3(**B**), (**B**)C1–H8(**E**), (**E**)C2–H6(**C**), (**C**)C1–H6(**D**) and (**D**)C1–H3(**A**), as well as between anomeric protons and glycosylated carbon atoms of adjacent sugar residues: (**A**)H1–C3(**B**), (**B**)H1–C8(**E**), (**C**)H1–C6(**D**) and (**D**)H1–C3(**A**).

**Figure 3:**
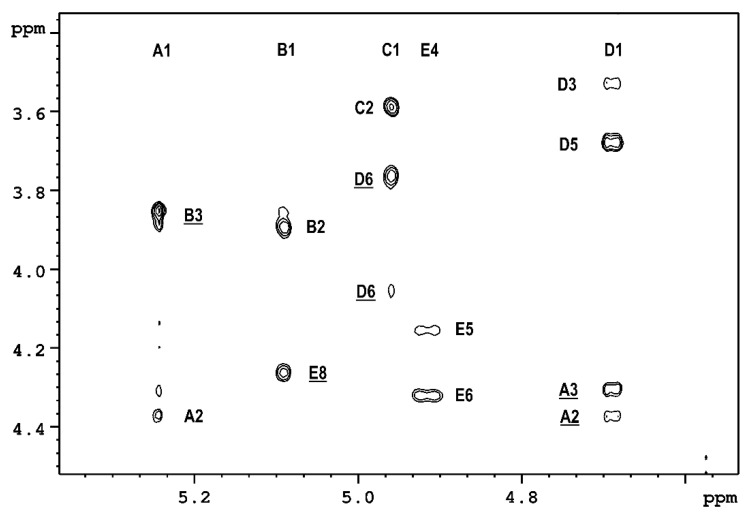
The section of ROESY spectrum of the OPS isolated from *P. parmentieri* SCC3193.

These findings were supported by the ^1^H,^1^H ROESY spectrum (Figure 3), which showed the *inte*r-residual proton contacts between A-1/B-3, B-1/E-8, **C**-1/**D**-6 and **D**-1/**A**-3.

Structural studies carried out for a polysaccharide isolated from the strain *P. parmentieri* IFB5432 provided practically the same information. Thus, the results of compositional analyses and NMR data allowed us to identify the structure of the OPS from *P. parmentieri* SCC3193 and *P. parmentieri* IFB5432 as:
→3)-β-d-Gal*f*-(1→3)-α-d-Gal*p*-(1→8)-β-Pse4Ac5Ac7Ac-(2→6)-α-d-Glc*p*-(1→6)-β-d-Glc*p*-(1→**
A
****
B
****
E
****
C
****
D
**

In summary, the structures of the O-polysaccharides isolated from two strains of bacterial phytopathogens, namely *P. parmentieri* SCC3193 and *P. parmentieri* IFB5432, were elucidated in this work. Both polysaccharides were identical and consisted of: →3)-Gal*f*, →3)-Gal*p*, two residues of →6)-Glc*p* and a very rare residue of 5,7-diamino-3,5,7,9-tetradeoxy-l-glycero-l-manno-non-2-ulosonic acid substituted in position C-8. To the best of our knowledge, no identical structure of an O-polysaccharide repeating unit has been previously described in any *Pectobacteriaceae* or other bacterial strain. The residue of 5,7-diamino-3,5,7,9-tetradeoxy-l-glycero-l-manno-non-2-ulosonic acid was reported earlier in the repeating unit of the polysaccharide isolated from *Shigella boydii* type 7 [20]. However, in the polysaccharide described in this work, an acetyl group was attached to the amino group at the C-7 carbon atom, while in the polysaccharide isolated from *Shigella boydii* type 7, there was a butyric acid residue instead [20].

To date, only several structures of O-polysaccharides of the LPS of *Pectobacterium* spp. Have been previously identified. These OPS molecules were characterized in *P. atrosepticum* [21,22], *P. parmentieri* (at that time classified as *P. wasabiae*) [18], *P. carotovorum* [23] and *P. brasiliense* [24]. Three other OPS from pectinolytic bacteria from the closely-related genus *Dickeya* were also structurally identified. In more detail, an identical OPS structure was detected in four strains of *D. solani* and a *D. dadantii* 3937 strain [25,26], while a different molecule was noted in *D. aquatica* IFB0154 [27]. All things considered, there were no significant structural similarities between the polysaccharides isolated from the herein described *P. parmentieri* SCC3193 and IFB5432 strains and the OPS characteristic of the aforementioned bacterial strains.

As the OPS of *P. parmentieri* SCC3193 and IFB5432 turned out to be structurally different from the only previously described OPS molecule in this species (which was characteristic of the *P. parmentieri* IFB5427 and IFB5408 strains), we started searching for an explanation for this phenomenon, both on the genomic and phenotypic levels. Special emphasis was put on features associated with the virulence of this plant pathogenic bacterium. Notably, in the following biodiversity-associated experiments, we also included a *P. parmentieri* IFB5441 strain, as we had already gathered evidence for a third structurally diverse OPS molecule, which is characteristic of that specific isolate (manuscript in preparation).

### 2.2. Genomic Structure of P. parmentieri Strains Containing Diverse OPS

In our former study, a genome-wide analysis based on repetitive extragenic palindromic sequence polymerase chain reaction (REP-PCR) was conducted on 85 *P. parmentieri* strains of Polish origin. Five REP genomic profiles were differentiated with pattern I being the most prevalent (>44%) and characteristic of SCC3193 [10]. Interestingly, the herein shown *P. parmentieri* SCC3193 and IFB5432 strains did not exhibit the same REP profiles (SCC3193 no. I and IFB5432 no. III) [9]. On the other hand, the *P. parmentieri* IFB5427 and IFB5408 strains of an identical but diverse OPS structure from the above-listed strains did share the most frequently observed REP profile, no. I. Curiously, the *P. parmentieri* IFB5441 that possesses a third unique OPS molecule also differed from the before-mentioned strains in terms of the exhibited REP profile (no. II) [9].

To further dig into the whole genome-associated diversity among *P. parmentieri* strains, we previously de novo sequenced the genomes of 10 strains of Polish origin and two isolated in Belgium [10]. Pangenome analysis, which allows us to reveal how many genes are unique or shared among the included strains, disclosed a rich pool of accessory (1468) and unique (1847) genetic units, alongside a relatively small core pangenome fraction (3706). Among the *P. parmentieri* strains whose OPS structures have been revealed, the genome of *P. parmentieri* SCC3193 was reported to possess the highest number of unique genes (346), in contrast to IFB5408, which showed the lowest number of unique genes (45). We did not observe a relationship between the number of unique genes and the exhibited strain-specific OPS molecule.

In more detail, the genes encoding proteins involved in the biosynthesis of LPS were analyzed during pangenome-oriented studies on *P. parmentieri* species [10]. This research revealed that the genes responsible for the biosynthesis of LPS were observed in the accessory pangenome fraction, which means that they were not present in all the genomes of the 15 strains analyzed. Precisely, the IFB5408 strain possessed additional, strain-specific copies of the genes encoding LPS ABC transporter substrate-binding protein LptA, LPS export ABC transporter periplasmic protein LptC and also an additional copy of an LPS ABC transporter ATP-binding protein [10].

Thus, we herein decided to evaluate the genome-wide sequence accord between the five *P. parmentieri* strains characterized by their already established OPS structure. Interestingly, the highest similarity-related values according to all the applied methods, i.e., BLAST+ calculation of ANI (ANIb; Table 2), MUMmer calculation of ANI (ANIm; Table 3) and calculation of the correlation indexes of tetra-nucleotide signatures (Tetra; Table 4), were noted for *P. parmentieri* strains IFB5408 and IFB5427 that share an identical OPS structure [18]. On the contrary, we did not make such observation for the *P. parmentieri* SCC3193 and IFB5432 strains, which also possess the same OPS structure, though different from that of the IFB5408 and IFB5427 isolates. It is worth acknowledging that the lowest values, i.e., 98.60 and 98.68, in ANIb analysis (Table 2) were noted for pairwise comparisons involving the *P. parmentieri* IFB5441 strain (with IFB5427 and IFB5408, respectively) of a diverse OPS structure. Concerning ANIm, the most inferior value of 99.01 (Table 3) also involved strains yielding different OPS molecules, though this time, they were IFB5408 and IFB5432. In terms of the calculation of Tetra correlation indexes, the genomes of SCC3193 and IFB5427 (also diverse OPS producers), with a value of 0.99967 (Table 4), displayed the lowest similarity rate in this assay.

It is worth noting that the herein reported lowest ANIb value turned out to be inferior to the ANI reported by Zoledowska et al. [10], even though the previous study included a higher number of *P. parmentieri* genomes (15). This result should be interpreted in the context of diverse bioinformatics tools applied in the juxtaposed research. In spite of diverse methodologies, the former research of Zoledowska et al. [10]—like the current study—revealed one of the lowest ANI values in comparisons involving strains producing different OPS structures, e.g., IFB5441 in relation either to IFB5408 (98.98) or IFB5427 (98.98), or IFB5432 juxtaposed either to IFB5408 (98.95) or IFB5427 (98.99). It is also worth discussing a *P. parmentieri* phylogeny computed basing on blast all against all protein sequences in relation to the displayed OPS structure. The formerly reported relatedness suggests either the closest genetic distance is between IFB5427 and IFB5408 or IFB5441 and IFB5432. The first relatedness finds confirmation in the herein reported OPS-related background, contrarily to the second cladding. All things considered, the most complex picture of the genomic relationships between certain strains arises after applying the greatest number of comparative genomics tools.

### 2.3. Analysis of the Phenotypic Features and Ability to Cause Disease Symptoms of P. parmentieri Strains Exhibiting Different OPS Structures

To investigate whether there is a possible relationship between the chemical composition of the OPS molecules and the phenotypic features of *P. parmentieri* strains, we compared a broad panel of biochemical characteristics and the most important virulence factors among all five studied *P. parmentieri* strains. Here, we discuss the former results of pectinase, cellulase and protease activities, siderophore production, the ability to swim and macerate potato tissue, as established for all the herein investigated *P. parmetieri* strains [9] in an OPS-associated context. Furthermore, additional metabolism, pathogenicity and growth condition-related assays were performed. They involved establishment of API 20E biochemical profiles, estimation of bacterial ability to macerate chicory leaves and evaluation of microbial sensitivity to diverse temperatures and elevated salinity.

All of the investigated *P. parmentieri* strains were capable of growing at 20 °C and 28 °C but failed to grow at 37 °C and in the presence of 5% NaCl. All the tested *P. parmentieri* strains exhibited similar biochemical features in the API 20E set (Table 5). In detail, each of the *P. parmentieri* strains was unable to metabolize sorbitol or inositol, did not produce arginine dihydrolase, lysine decarboxylase, ornithine decarboxylase, H_2_S, urease, tryptophan deaminase, indole or gelatinase and was incapable of citrate degradation.

**Figure 4:**
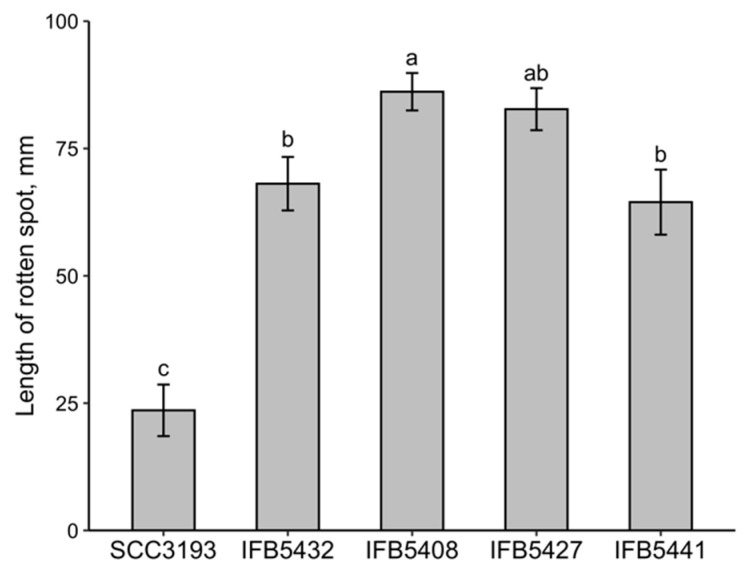
Comparison of the ability of *P. parmentieri* strains to cause disease symptoms on chicory leaves. Means ± SE of the lengths of the rotten spots on chicory leaves are depicted. Three independent experiments with 11 technical replications were conducted. Means marked with different letters (a, b, c) are significantly different according to the Kruskal–Wallis test followed by a posthoc analysis applying Fisher’s least significant criterion at *p* < 0.05 (H = 67.6, df = 4, *p* < 0.001).

The physiological and biochemical characteristics of the studied *P. parmentieri* strains are consistent with the previous reports for *P. parmentieri* SCC3193 [2,4] in terms of the inability of this strain to grow at 37 °C, in 5% NaCl or to assimilate melibiose. Previous studies showed that the following *P. parmentieri* strains, SCC3193, IFB5408, IFB5427, IFB5432 and IFB5441, do not display significant differences in their motility, pectinases or cellulases activities, but IFB5432 and IFB5441 show lower siderophore production capacity than both IFB5408 and IFB5427 [9]. Interestingly, the *P. parmentieri* SCC3193 strain revealed no protease activity nor siderophore production [9]. Subsequently, the association between the ability of *P. parmentieri* to macerate plant tissues and the exhibited composition of the OPS molecule was examined. Here, the *P. parmentieri* SCC3193 strain turned out to macerate chicory leaves in a significantly less efficient manner (at *p* < 0.05) than the other *P. parmentieri* strains IFB5432, IFB5427, IFB5408 and IFB5441 (Figure 4). In contrast, our previous research showed that all *P. parmentieri* strains of interest, i.e., SCC3193, IFB5432, IFB5408, IFB5427 and IFB5441, degraded potato tissue to a similar extent [9]. Putatively, the lower chicory leaf maceration capacity of SCC3193 may be associated either with the presence of different plant metabolites in this tissue or bacterium-related factors such as a lack of protease activity or differences in the exhibited OPS molecule.

Several studies have indicated that the composition or size of the O-antigen might be a reliable indicator of the bacterial virulence potential. For instance, *D. aquatica* strain IFB0154 isolated from water had a different OPS structure (which contained fucose and rhamnose) than that of the other investigated *Dickeya* spp. strains isolated from diseased plants (in their case the OPS consisted of 6-deoxyaltrose repeating units) [25,27]. Notably, *D. aquatica* IFB0154 was reported to be much less efficient in rotting potato and chicory tissues than the other members of the *Dickeya* genus [28].

Although in the present work, we did not observe an apparent relationship between the virulence of the tested *P. parmentieri* strains and the exhibited strain-specific OPS molecule, the current and forthcoming studies on the LPSs of different soft-rotting phytopathogens in broad phenotypic and genomic contexts will improve our understanding of the mechanisms of pathogenesis in plant pathogenic bacteria.

The obtained results constitute a very good basis for examining the importance of LPS in the infection process caused by an economically significant soft-rotting bacterium, namely *P. parmentieri*. Further work involving, e.g., the construction of deletion mutants in genes encoding different constituents of the LPS synthesis machinery may allow us to unveil the role of LPS in the virulence of the studied phytopathogen.

## 3. Materials and Methods

### 3.1. Origin and Biomass Production of P. parmentieri SCC3193 and IFB5432 for the OPS Analysis

*P. parmentieri* SCC3193 (formerly *Erwinia carotovora* subsp. *carotovora* SCC3193, *P. carotovorum* subsp. *carotovorum* SCC3193 or *P. wasabiae* SCC3193 [2,3,4]) was isolated from potato in Finland in the 1980s and received from Prof. Minna Pirhonen (University of Helsinki, Finland). Strain IFB5432 was isolated from symptomatic potato tubers in Poland in 2013 and deposited in the bacterial collection of the Intercollegiate Faculty of Biotechnology, University of Gdansk and Medical University of Gdansk (IFB UG & MUG) [9].

To acquire approx. 50 g of the frozen bacterial biomass per strain, P. parmentieri SCC3193 and IFB5432 were grown on trypticase soy agar plates (TSA, Oxoid, Basingstoke, UK) at 28 °C for 24 h. Then, single bacterial colonies were utilized to inoculate 200 mL flasks with trypticase soy broth (TSB, Oxoid, Basingstoke, UK) medium, which were then incubated at 28 °C with 130 rpm shaking for 24 h. Each of these flasks was used afterward for inoculation of 2000 mL TSB, which was subjected to 130 rpm shaking at 28 °C lasting for 48 h. Subsequently, bacterial cells were harvested with the use of a Sorvall centrifuge (Thermo Fisher Scientific, Waltham, MA, USA) (16,915× *g* for 10 min) and stored at −25 °C for further OPS-oriented analyses.

### 3.2. Determination of the OPS Structure of P. parmentieri SCC3193 and IFB5432 Strains

#### 3.2.1. LPS Extraction and Purification

The classical phenol-water extraction method was used to isolate LPS (83.0 mg and 86.1 mg) from the lyophilized bacterial cells (10.1 g and 13.9 g) of *P. parmentieri* SCC3193 and IFB5432, respectively [29]. Briefly, the bacterial cells were suspended in 200 mL of hot water and hot phenol (1:1, *v*/*v*). The mixture was stirred at 65 °C for 30 min and centrifuged (7012× *g*, 20 min, 4 °C). The aqueous phase was dialyzed against distilled water and lyophilized. Nucleic acids were precipitated with 40% ethanol at pH 4. Ethanol was removed by dialysis and the sample was lyophilized, dissolved in 15 mL of 0.5 M NaCl and after addition of 150 mL of cold ethanol (99.8%), was placed in a refrigerator (4 °C, 1 h). Finally, the pellet obtained after centrifugation was suspended in distilled water, dialyzed (24 h) and freeze-dried [25].

#### 3.2.2. Isolation of OPS

The LPS was hydrolyzed with 1% acetic acid (100 °C, 1 h), and the lipid A was removed by centrifugation. The supernatant was freeze-dried, dissolved in water and fractionated by GPC on a Bio-Gel P-100 (Bio-Rad; Hercules, CA, USA) column (100 × 0.9 cm) with water as the eluent. The separation was monitored by the RI-2031 Jasco differential refractometer detector (JASCO, Tokyo, Japan). The collected fractions were freeze-dried [18]. Finally, 5.0 mg (SCC3193) and 8.2 mg (IFB5432) of high-molecular-mass OPS were obtained.

#### 3.2.3. Chemical Analysis

The monosaccharide composition was determined using sugar analysis [27]. The samples were hydrolyzed with trifluoroacetic acid (2M TFA, 2 h at 120 °C), reduced with sodium borohydride (NaBH_4_) and acetylated with acetic anhydride in the presence of sodium acetate (120 °C, 2 h). The obtained derivatives were analyzed using the GLC and GLC-MS techniques.

The substitution positions of the monosaccharides were assigned via methylation analysis. Dimethyl sulfoxide (DMSO) was used as a solvent in the presence of solid potassium hydroxide, and then the methyl iodide was added. Methylated polysaccharides, after removing excess methyl iodide from the nitrogen stream, were extracted with chloroform. Then, the methylated O-polysaccharides were hydrolyzed, reduced and acetylated, as was described above for the sugar analysis. Finally, the obtained derivatives were analyzed by GLC and GLC-MS [18].

The absolute configuration of the monosaccharides was determined by analyses of glycoside derivatives. The polysaccharides were hydrolyzed (2M TFA, 2 h, 120 °C) and subsequently subjected to reaction with (*S*)-(+)-butan-2-ol in the presence of TFA (6 h, 105 °C). The obtained acetate derivatives of butyl glycosides were analyzed by GLC.

#### 3.2.4. GLC and GLC-MS Analyses

GLC analyses were performed using a gas chromatograph FISONS GC 8000 (Fisons, Ipswich, UK) equipped with a flame ionization detector and a capillary column Rtx-5 (30 m) for sugar and methylation analyses, as well as a capillary column Rtx-2330 (60 m) for the acetylated 2-butyl glycosides. The temperature program was 120–260 °C, 4 °C/min, 20 min isotherm at 260 °C. For all GLC-MS analyses, a Shimadzu GC-MS-QP2010SE system (Shimadzu, Kioto, Japan) equipped with a capillary column Rtx-5 (30 m) was used (program temperature: 120–260 °C, 4 °C/min, 20 min isotherm at 260 °C; EI ionization −70 eV; ion source temperature −220 °C; recorded mass range: 43–550 m/z).

#### 3.2.5. NMR Spectroscopy

NMR spectra were recorded using a Bruker Avance III 700 MHz spectrometer (Bruker, MA, USA) at 48 °C. All 1D (^1^H) and 2D ^1^H, ^1^H homonuclear (double quantum filtered correlation spectroscopy [DQF-COSY], total correlation spectroscopy [TOCSY] and rotation frame nuclear Overhauser effect spectroscopy [ROESY]), as well as 2D ^1^H, ^13^C heteronuclear (heteronuclear single quantum coherence [HSQC], heteronuclear multiple bond correlation [HMBC] and heteronuclear single quantum coherence–total correlation spectroscopy [HSQC–TOCSY]) experiments were recorded using standard Bruker pulse programs. Both polysaccharides (3.9 mg of SCC3193 and 8.2 mg of IFB5432) were dissolved in 1 mL of 99% D_2_O and freeze-dried to replace all exchangeable protons. The process was repeated twice, and the final sample was dissolved in 0.6 mL of 99.9% D_2_O. Chemical shifts were referenced to acetone (δ_H_ 2.225, δ_C_ 31.45).

### 3.3. Characterization of P. parmentieri Strains of the Already Deciphered OPS Structure

Apart from the *P. parmentieri* SCC3193 and IFB5432 strains, whose identical OPS chemical structure has been described for the first time in this work, the previously studied *P. parmentieri* IFB5427 and IFB5432 strains—displaying a different OPS molecule [18] to that currently investigated—were included in the following experiments. The fifth *P. parmentieri* IFB5441 strain, yielding a strain-specific OPS molecule (manuscript in preparation), was applied for comparative purposes.

#### 3.3.1. Whole-Genome Comparisons

The whole genome-based analyses of *P. parmentieri* strains whose OPS structures have been delineated to date involved pairwise genome comparisons computed with ANIb, ANIm and Tetra algorithms on the JSpecies web server [30]. For ANIb and ANIm, the percentages of the aligned sequences were also included.

#### 3.3.2. Phenotypic Assays

Overnight cultures of *P. parmentieri* in TSB were centrifuged for 10 min at 4779× *g*, washed twice and subsequently suspended in a sterile 0.85% NaCl solution to reach the turbidity of 0.5 on the McFarland scale (McF). Then, 2 μL of the prepared bacterial suspensions were used to inoculate the plates to test the growth of *P. parmentieri* strains at different temperatures (20, 28 and 37 °C) and the ability of these strains to grow on TSA supplemented with 5% NaCl. The growth assessments were performed after 48 h of incubation at the stated temperature. The experiments were repeated twice with three technical replicates.

Biochemical profiles of the *P. parmentieri* strains were acquired from an API 20E (bioMérieux Inc., Marcy-l’Étoile, France) assay, which was carried out according to the manufacturer’s instructions. The following biochemical characteristics: enzyme activities (β-galactosidase, arginine-dihydrolase, lysine decarboxylase, ornithine decarboxylase, urease, tryptophan deaminase and gelatinase); citrate utilization; production of sulfide, indole and acetoin; fermentation or oxidation of sugars (glucose, mannitol, inositol, sorbitol, rhamnose, sucrose, melibiose, amygdalin and arabinose) and nitrate reduction, were tested. The experiment was repeated twice.

The virulence of the studied *P. parmentieri* strains was evaluated by the ability of these strains to cause the rotting of chicory leaves as described in [31]. The chicory leaves were slightly wounded in the middle at the bacterial inoculation site. Eleven leaves were infected for each strain using 10 μL of a 0.5 McF bacterial suspension. In terms of the negative control sample, 10 μL of sterile 0.85% NaCl was used for inoculations instead of the bacterial suspension. The inoculated chicory leaves were placed in plastic bags on wet linen and incubated for 48 h at 28 °C. To estimate the disease severity, the lengths of the resultant rotten spots were measured. The experiment was repeated three times.

### 3.4. Statistical Analyses

Statistical analyses were performed using R statistical software [32] version 4.1.2 within RStudio statistical software version 1.4.1106. Shapiro–Wilk’s test was implemented to evaluate the normality of the data, while Levene’s test was applied to test the equality of variance. A non-parametric Kruskal–Wallis test and subsequent posthoc test applying Fisher’s least significant difference criterion were utilized. A *p*-value of less than 0.05 was considered significant for all the calculations.

## Figures and Tables

**Table 1:** ^1^H and ^13^C NMR data of the OPS isolated from *P. parmentieri* SCC3193. The glycosylated carbon atoms are underlined.

Residue [^1^*J*_C1-H1;_ ^3^*J*_H1-H2_ (Hz)]	Chemical Shifts of ^1^H and ^13^C [ppm]								
	H1 C1	H2 C2	H3 C3	H4 C4	H5 C5	H6 C6	H7 C7	H8 C8	H9 C9
→3)-β-D-Gal*f* (**A**) [172; <1.5]	5.241 110.71	4.373 81.25	4.303 85.77	4.199 83.39	3.932 71.87	3.722/3.686 64.14	-	-	-
→3)-α-D-Gal*p* (**B**) [171; 3.7]	5.088 98.06	3.892 68.56	3.854 78.64	4.138 70.52	4.209 71.96	3.756/3.756 62.36	-	-	-
→6)-α-D-Glc*p* (**C**) [172; 3.4]	4.959 99.27	3.592 72.84	3.737 74.45	3.410 71.39	3.809 71.87	3.928/3.709 64.78	-	-	-
→6)-β-D-Glc*p* (**D**) [161; 7.8]	4.689 103.29	3.328 74.29	3.528 77.19	3.581 70.49	3.676 75.74	4.051/3.762 66.79	-	-	-
→8)-β-Pse4Ac5Ac7Ac-(2→ (**E**) -*O*-Ac -*N*-Ac -*N*-Ac	- 174.38 - 174.50 - 175.57 - 174.13	- 102.33 1.956 23.48 2.008 23.21 2.014 21.76	2.489/1.784 34.09	4.911 70.59	4.325 47.12	4.157 72.59	4.325 54.15	4.267 74.13	1.293 14.19

**Table 2:** ANIb-based pairwise genome comparisons between the studied *P. parmentieri* strains.

	SCC3193	IFB5432	IFB5408	IFB5427	IFB5441
SCC3193	-	98.81 [90.29]	98.69 [90.50]	98.70 [89.58]	98.78 [90.14]
IFB5432	98.91 [93.22]	-	98.77 [93.27]	98.76 [93.14]	99.89 [97.47]
IFB5408	98.80 [93.00]	98.76 [92.98]	-	99.94 [98.41]	98.77 [92.94]
IFB5427	98.74 [90.64]	98.73 [91.22]	99.93 [96.57]	-	98.69 [91.25]
IFB5441	98.79 [91.61]	99.77 [96.05]	98.68 [91.73]	98.60 [91.70]	-

Jspecies was used for the calculations. The ANIb values are shown above the percentages of the aligned sequences depicted in parenthesis.

**Table 3:** ANIm-based pairwise genome comparisons between the studied *P. parmentieri* strains.

	SCC3193	IFB5432	IFB5408	IFB5427	IFB5441
SCC3193	-	99.13 [90.98]	99.06 [91.15]	99.06 [90.22]	99.11 [90.87]
IFB5432	99.13 [94.02]	-	99.01 [93.99]	99.03 [93.83]	99.90 [97.78]
IFB5408	99.06 [93.75]	99.01 [93.63]	-	99.99 [98.57]	99.02 [93.62]
IFB5427	99.06 [91.35]	99.03 [91.84]	99.99 [96.69]	-	99.04 [91.63]
IFB5441	99.12 [92.38]	99.91 [96.37]	99.02 [92.53]	99.04 [92.30]	-

Jspecies was used for the calculations. The ANIm values are shown above the percentages of the aligned sequences depicted in parenthesis.

**Table 4:** Correlation indexes of the tetra-nucleotide signatures computed for the studied *P. parmentieri* strains.

	SCC3193	IFB5432	IFB5408	IFB5427	IFB5441
SCC3193	-	0.99975	0.99973	0.99967	0.99975
IFB5432	0.99975	-	0.99987	0.99987	0.99991
IFB5408	0.99973	0.99987	-	0.9999	0.99984
IFB5427	0.99967	0.99987	0.9999	-	0.99979
IFB5441	0.99975	0.99991	0.99984	0.99979	-

Jspecies was used for the calculations.

**Table 5:** Biochemical features of the studied *P. parmentieri* strains as identified by API 20E.

Biochemical Feature	SCC3193	IFB5432	IFB5408	IFB5427	IFB5441
β-galactosidase activity	+	+	+	+	+
Arginine dihydrolase activity	-	-	-	-	-
Lysine decarboxylase activity	-	-	-	-	-
Ornithine decarboxylase activity	-	-	-	-	-
Citrate utilization	-	-	-	-	-
H_2_S production	-	-	-	-	-
Urease production	-	-	-	-	-
Tryptophane deaminase activity	-	-	-	-	-
Indole production	-	-	-	-	-
Acetoin production (VP)	-	-	-	-	-
Liquefaction of gelatin	-	-	-	-	-
Fermentation of glucose	+	+	+	+	+
Fermentation of mannitol	+	+	+	+	+
Fermentation of inositol	-	-	-	-	-
Fermentation of sorbitol	-	-	-	-	-
Fermentation of rhamnose	+	+	+	+	+
Fermentation of saccharose	+	+	+	+	+
Fermentation of melibiose	+	+	+	+	+
Fermentation of amygdalin	+	+	+	+	+
Fermentation of arabinose	+	+	+	+	+
NO_3_ reduction to NO_2_	+	+	+	+	+

“-” strain is capable of conducting the stated biochemical reaction, “+” strain is unable to conduct the stated biochemical reaction.

## Data Availability

Not applicable.

## Supplementary Materials

The following are available online at https://www.mdpi.com/article/10.3390/ijms23042077/s1.
