# Supplementary material for: Heterogenicity within the LPS Structure in Relation to the Chosen Genomic and Physiological Features of the Plant Pathogen Pectobacterium parmentieri
Source: Int J Mol Sci. 2022 Feb 14;23(4):2077. doi: 10.3390/ijms23042077 (PMC8879369; doi:10.3390/ijms23042077)

Supplementary materials

Heterogenicity within the LPS Structure in Relation to the Chosen Genomic and Physiological Features of the Plant Pathogen *Pectobacterium parmentieri*

Karolina Ossowska [1], Agata Motyka-Pomagruk [2], Natalia Kaczyńska [2], Agnieszka Kowalczyk [1], Wojciech Sledz [2], Ewa Lojkowska [2] and Zbigniew Kaczyński [1,*]

[1] Faculty of Chemistry, University of Gdansk, 63 Wita Stwosza, 80-308 Gdansk, Poland; karolinaoss@wp.pl (K.O.); agnieszka.kowalczyk@ug.edu.pl (A.K.)

[2] Intercollegiate Faculty of Biotechnology, University of Gdansk and Medical University of Gdansk, 58 Abrahama, 80-307 Gdansk, Poland; agata.motyka-pomagruk@ug.edu.pl (A.M.-P.); natalia.kaczynska@ug.edu.pl (N.K.); wojciech.sledz@biotech.ug.edu.pl (W.S.); ewa.lojkowska@biotech.ug.edu.pl (E.L.)

* Correspondence: zbigniew.kaczynski@ug.edu.pl

Figure S1. The HSQC spectrum of the OPS isolated from *P. parmentieri* SCC3193

Figure S2. The TOCSY spectrum of the OPS isolated from *P. parmentieri* SCC3193

Figure S3. The COSY spectrum of the OPS isolated from *P. parmentieri* SCC3193

Figure S1. The HSQC spectrum of the OPS isolated from *P. parmentieri* SCC3193.

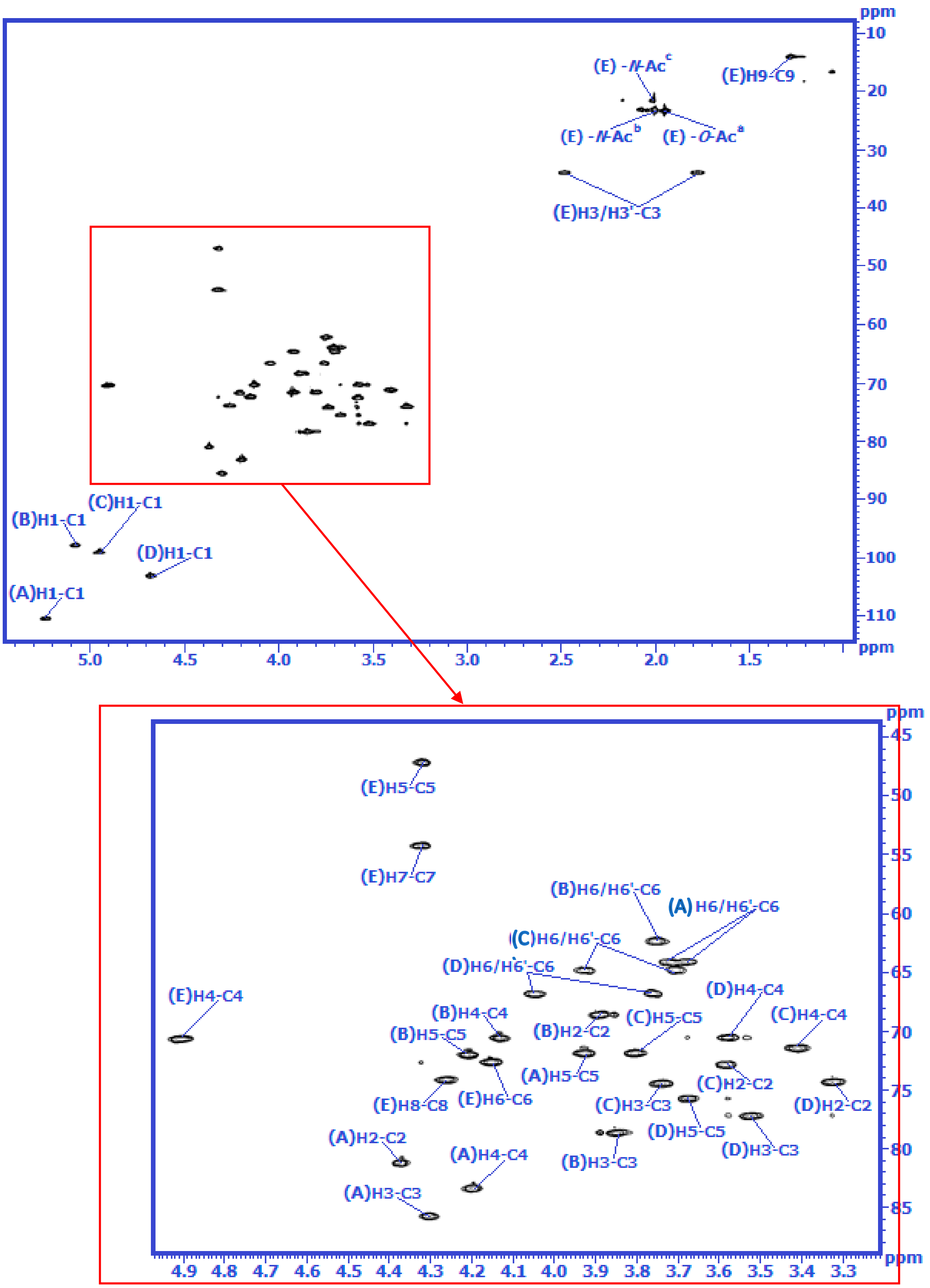

Figure S2. The TOCSY spectrum of the OPS isolated from *P. parmentieri* SCC3193.

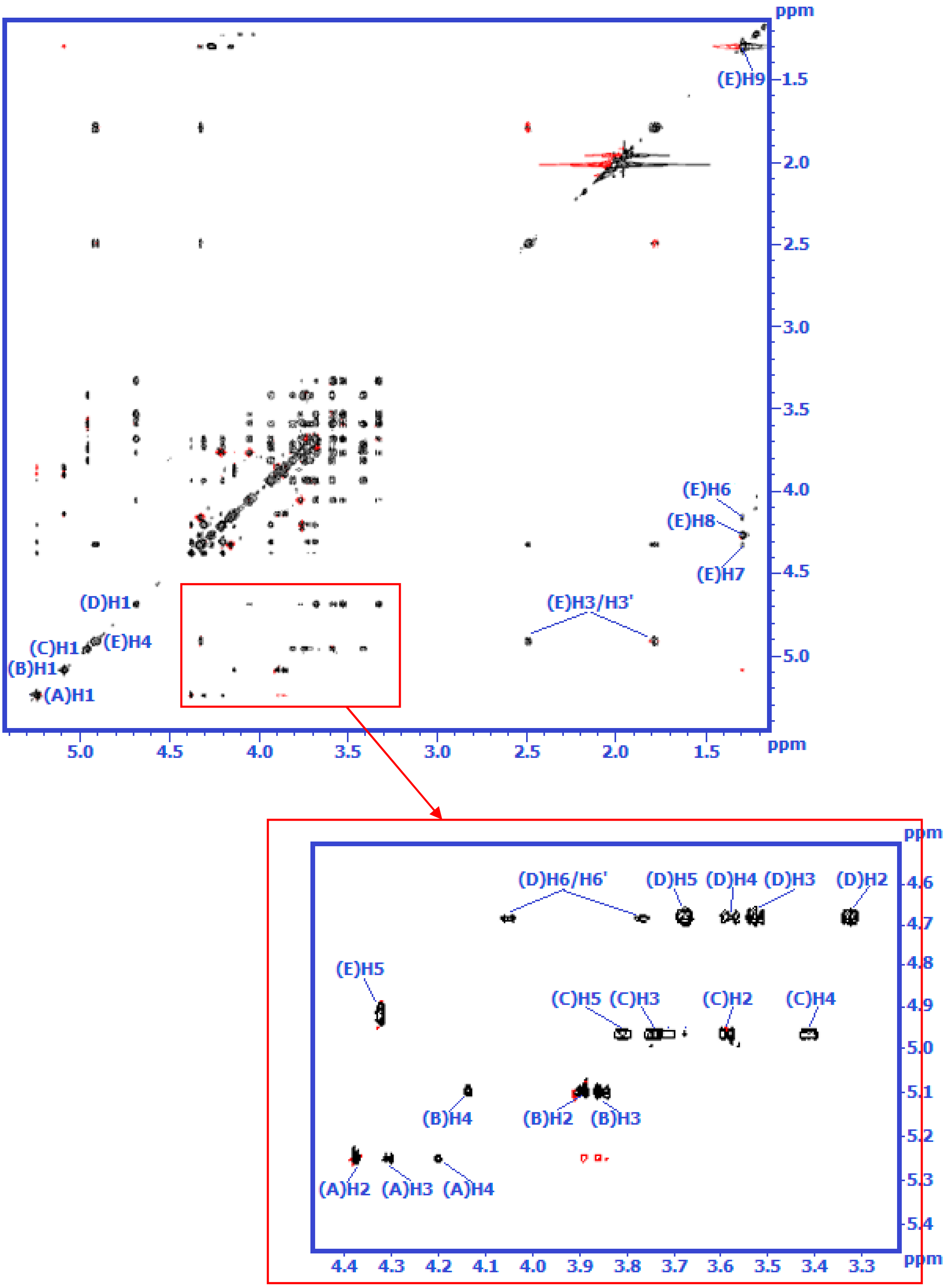

Figure S3. The COSY spectrum of the OPS isolated from *P. parmentieri* SCC3193.

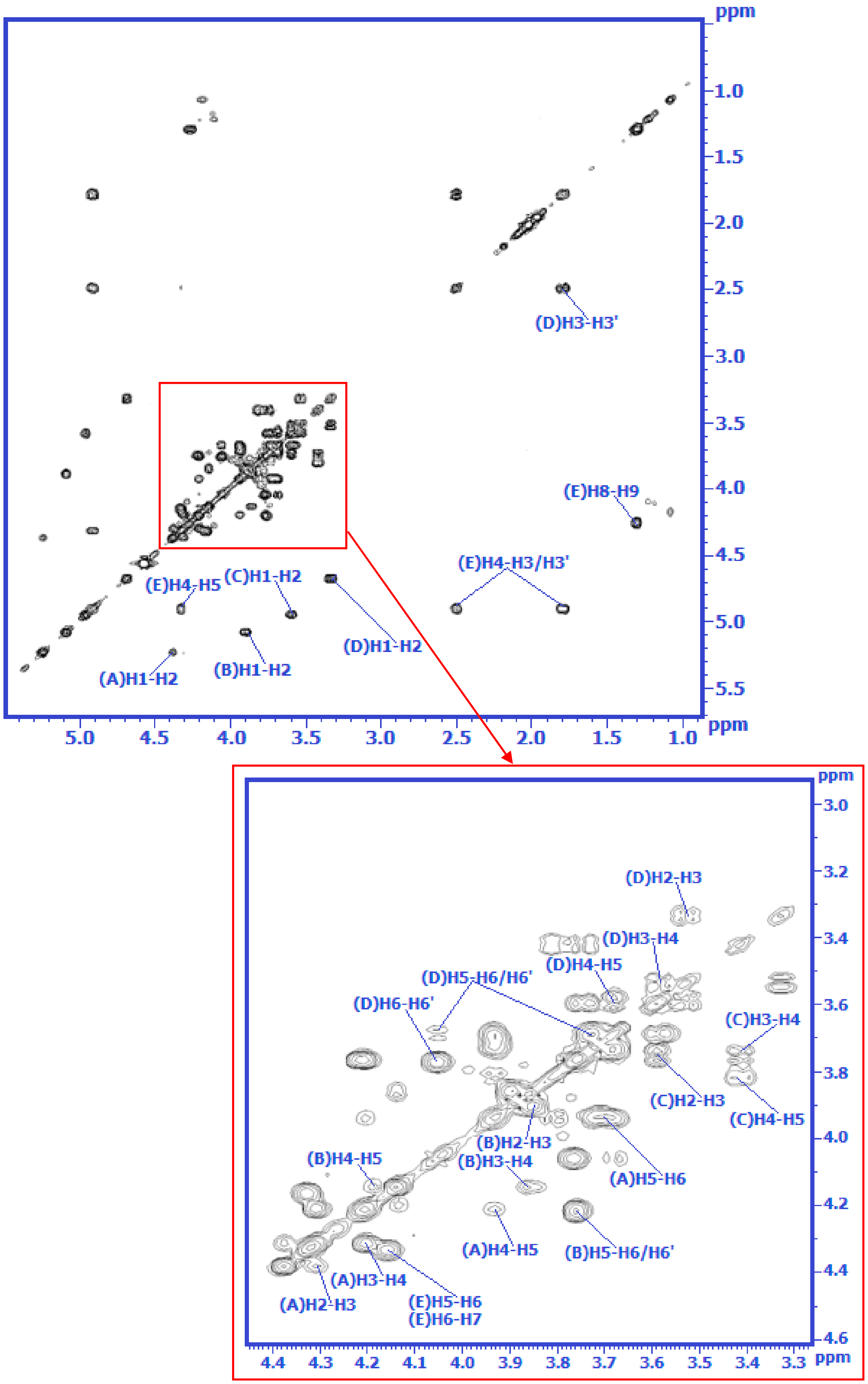

Supplement: Supplementary file 1 [file ijms-23-02077-s001.zip › ijms-1558085-supplementary.pdf]
